# Microbiological etiology of endogenous and exogenous postprocedural endophthalmitis: a 5-year German federal state study

**DOI:** 10.1007/s15010-026-02763-5

**Published:** 2026-03-25

**Authors:** Colya N. Englisch, Tim Berger, Fabian N. Fries, Alexander Halfmann, Markus Bischoff, Philip Wakili, Annekatrin Rickmann, Boris V. Stanzel, Eugen Reifschneider, Marc A. Macek, Alaa Din Abdin, Shady Suffo, Loay Daas, Karl T. Boden, Peter Szurman, Berthold Seitz, Sören L. Becker, Clara E. Englisch, Núria Pérez Guerra

**Affiliations:** 1 Institute of Medical Microbiology and Hygiene, Center for Infectious Diseases, Saarland University, Kirrberger Strasse 100, 66421 Homburg, Germany; 2 Eye Clinic Sulzbach, Knappschaft Hospitals Saar, 66280 Sulzbach, Germany; 3 Department of Experimental Ophthalmology, Saarland University, 66421 Homburg, Germany; 4 Department of Ophthalmology, Saarland University Medical Center, 66421 Homburg, Germany

**Keywords:** Endophthalmitis, Endogenous, Exogenous, Pathogen spectrum, Antimicrobial susceptibility, Culture, Polymerase chain reaction, MALDI-TOF mass spectrometry

## Abstract

**Purpose:**

To investigate the microbiological etiology of endogenous and exogenous postprocedural infectious endophthalmitis in patients referred to two tertiary eye care centers in the German federal state of Saarland over a five-year period (2019–2023).

**Methods:**

We retrospectively analyzed all cases of endophthalmitis treated at two ophthalmological centers in southwest Germany between January 1, 2019, and December 31, 2023. Included were only those cases for which vitreous aspirate specimens were subjected to microbiological analysis. Cases of exogenous endophthalmitis secondary to other ocular or periocular infections (e.g., microbial keratitis) or trauma were excluded. Microbiological pathogen identification was performed using Gram stain microscopy, conventional bacterial and fungal cultures, as well as broad-range bacterial and fungal polymerase chain reaction (PCR), followed by sequencing in PCR-positive samples.

**Results:**

A total of 126 endophthalmitis vitreous aspirate samples from 126 patients with a mean age of 73.3 ± 11.4 years were included. The majority of endophthalmitis cases occurred after intravitreal injection (55.6%), followed by previous cataract surgery (23.8%), pars plana vitrectomy (5.6%), and glaucoma surgery (4.8%). Only 7.9% of all cases were endogenous. Overall, pathogens were identified in 58.7% (n = 74) of all cases, with culture yielding a higher detection rate compared to PCR (53.2% *versus* 43.5%). *Staphylococcus epidermidis* was most commonly detected (n = 43; 58.1%), followed by *Staphylococcus aureus* (n = 7; 9.5%) and *Enterococcus faecalis* (n = 5; 6.8%). Postinjection and postcataract endophthalmitis tended to be associated with positive bacterial culture results, whereas endogenous endophthalmitis was more frequently associated with negative bacterial culture results (*p* = 0.06). Fungal endophthalmitis was detected in three samples, of which the endogenous cases were caused by *Candida albicans* (n = 2). Bacterial multidrug resistance was rare, with only a single detected methicillin-resistant *S. aureus* strain. All Gram-positive bacteria were susceptible to vancomycin.

**Conclusion:**

Our results confirm *S. epidermidis* to be the most common endophthalmitis-causing pathogen. No single microbiological diagnostic technique would have identified all cases of infection, thus calling for combined approaches that include culture and molecular testing.

## Introduction

Infectious endophthalmitis is a serious ocular disease that frequently leads to significant and irreversible vision deterioration [1]. In terms of exogenous causes, infectious endophthalmitis can occur after trauma, surgery (e.g., cataract surgery), and medical interventions (e.g., intravitreal injections), or in the context of other ocular (e.g., keratitis) or periocular infections [1]. Endogenous endophthalmitis is characterized by the absence of direct causative ophthalmological factors and may occur as a complication of systemic infections such as *Staphylococcus aureus* bacteremia [2]. Gram-positive bacteria (e.g., *Staphylococcus epidermidis*, oral streptococci, *S. aureus*, *Cutibacterium acnes*, etc.) are reported as the leading pathogens in infectious endophthalmitis, followed by Gram-negative bacteria (e.g., *Enterobacterales*, *Pseudomonas aeruginosa*), and fungi (e.g., *Candida* species) [3]. In-depth microbiological investigations are important to understand the etiology, the pathogen spectrum, and disease dynamics over shorter and longer time periods. Although several studies investigating regional pathogen spectra of microbial endophthalmitis have been published, recent data are scarce with regard to the etiology of endophthalmitis in Western European countries.

The aim of this study was to investigate the microbiological spectrum of endogenous and exogenous postprocedural endophthalmitis in patients presenting to two tertiary eye care centers in the German federal state of Saarland over a five-year period (2019–2023). A secondary aim was to study the diagnostic accuracy of different microbiological techniques (*i.e*. microscopy, culture, polymerase chain reaction [PCR] with subsequent sequencing) when being applied to vitreous samples in patients with endophthalmitis.

## Methods

This retrospective study was carried out in the two major ophthalmological hospitals of the Saarland area, i.e., Eye Clinic Sulzbach, Knappschaft Hospitals Saar in Sulzbach, and the Department of Ophthalmology at Saarland University Medical Center in Homburg. Saarland is one of 16 federal states in Germany, with a population of ~ 1 million and an area of 2.570 km^2^. We recorded all cases of endophthalmitis that were documented in these hospitals between 1st of January 2019 and 31st of December 2023, if vitreous aspirate samples had been sent for microbiological diagnostics.

Cases of exogenous endophthalmitis that developed during the course of other ocular or periocular infections (e.g., endophthalmitis secondary to keratitis) or trauma were excluded. Exogenous postprocedural endophthalmitis was recorded after occurrence of typical endophthalmitis signs such as blurred vision, conjunctival hyperemia and chemosis, corneal edema, or ocular pain following intraocular surgery. Endogenous endophthalmitis was diagnosed in the absence of the above defined exogenous causes. Cases were identified retrospectively through the institutional microbiology laboratory database based on vitreous specimens submitted for suspected endophthalmitis. The laboratory information system did not consistently differentiate between vitreous samples obtained via pars plana vitrectomy and those obtained via vitreous tap. Hence, this distinction could not be reliably assessed in all cases. Anterior chamber aspirates were not considered for this analysis. Demographic data included age, sex, and the primary ophthalmological diagnosis. Dates of sampling at the tertiary eye care centers and diagnostics at the Institute of Medical Microbiology and Hygiene, Saarland University Medical Center were recorded.

This study was performed in line with the principles of the Declaration of Helsinki and approved by the local Institutional Review Board (Ethikkommission bei der Ärztekammer des Saarlandes, 128/25 and 50/22).

Pathogen identification was achieved using a series of different diagnostic tests: (i) Gram stain microscopy for an initial identification of bacteria and yeasts (microscopy was omitted in isolated cases with a very small sample volume); (ii) bacterial and fungal culture using specific agar media (e.g., tryptic soy blood agar, chocolate agar), with matrix-assisted laser desorption/ionization time-of-flight (MALDI-TOF) mass spectrometry being used for identification of culture-grown colonies; and (iii) broad-range bacterial (i.e., 16S) and fungal (internal transcribed spacer [ITS] region) PCR, which was followed by specific Sanger sequencing in case of positive test results. Antimicrobial susceptibility testing was performed in accordance with recommendations put forth by the European Committee on Antimicrobial Susceptibility Testing (EUCAST) in its annually revised version.

Statistical analysis was performed using the GraphPad Prism software package (Version 10.1.0). Results are presented using mean ± standard deviation (SD) and range (minimum–maximum). Fisher’s exact test was used to assess independence in contingency tables. Spearman correlation was performed to investigate the strength and direction of potential relationships. Diagnostic agreement between culture and PCR was assessed using Cohen’s kappa index. *p*-values (*p*) were two sided and considered statistically significant when < 0.05.

## Results

Over the 5-year period, endophthalmitis vitreous aspirate samples were collected from 126 eyes of 126 patients. There were more males than females (67 *vs.* 59) and the mean age was 73.3 ± 11.4 years (range 42–92). Endophthalmitis related to previous intravitreal injection occurred in 55.6%, followed by cases after cataract surgery in 23.8%. Endogenous endophthalmitis accounted for 7.9% of all cases. Details are presented in Table 1.

**Table 1:** Distribution of exogenous postprocedural and endogenous endophthalmitis cases in Saarland, southwest Germany, 2019–2023

Etiology of endophthalmitis	Number (%)
Intravitreal injection	70 (55.6%)
Cataract surgery	30 (23.8%)
Endogenous	10 (7.9%)
Pars plana vitrectomy	7 (5.6%)
Glaucoma surgery	6 (4.8%)
Corneal transplantation	3 (2.4%)

Overall, microbiological examinations revealed at least one pathogen in 58.7% (n = 74) of all endophthalmitis cases. Three Gram-negative and three fungal cases were identified (Table 2).

**Table 2:** Pathogen distribution among 74 cases of microbiologically confirmed endophthalmitis (employing culture and PCR), including five cases with two concurrent bacterial species, in Saarland, southwest Germany, 2019–2023

Gram-positive bacteria	Gram-negative bacteria	Fungi
*Staphylococcus epidermidis* (n = 43)	*Haemophilus influenzae* (n = 2)	*Candida albicans* (n = 2)
*Staphylococcus aureus* (n = 7)	*Neisseria subflava* (n = 1)	*Aspergillus fumigatus* (n = 1)
*Enterococcus faecalis* (n = 5)		
*Streptococcus oralis* (n = 3)		
*Streptococcus pseudopneumoniae* (n = 2)		
*Streptococcus mitis* (n = 1)		
*Streptococcus agalactiae* (n = 1)		
*Streptococcus vestibularis* (n = 1)		
*Streptococcus gordonii* (n = 1)		
*Staphylococcus hominis* (n = 1)		
*Staphylococcus capitis* (n = 1)		
*Staphylococcus lugdunensis* (n = 1)		
*Staphylococcus haemolyticus* (n = 1)		
*Actinomyces neuii* (n = 1)		
*Kocuria rhizophila* (n = 1)		
*Dermacoccus nishinomiyaensis* (n = 1)		
*Paenibacillus macerans* (n = 1)		
*Bacillus circulans* (n = 1)		

Bacterial culture was positive in 53.2% (n = 67). Among these cases, a single species was detected in 92.5% (n = 62), and two concurrent species in 7.5% (n = 5, [i] *Streptococcus oralis* + *Neisseria subflava*, [ii] *S. oralis* + *Streptococcus mitis*, [iii] *S. epidermidis* + *Streptococcus gordonii*, [iv] *S. epidermidis* + *Kocuria rhizophila*, [v] *S. epidermidis* + *Streptococcus vestibularis*). Among the bacterial isolates detected by culture, 59.7% (n = 40) showed *S. epidermidis*, followed by *S. aureus* in 10.5% (n = 7) and *Enterococcus faecalis* in 7.5% (n = 5).

Bacterial PCR was positive in 43.5% (n = 57) and negative in 56.5% (n = 74) of all cases. The diagnostic agreement between bacterial PCR and culture was substantial, as assessed by Cohen’s kappa index (Kappa = 0.65, 95% CI 0.53–0.78, Table 3).

**Table 3:** Diagnostic agreement between bacterial culture and polymerase chain reaction (PCR) results for the detection of endophthalmitis-causing bacteria, including five cases with two species, in Saarland, southwest Germany, 2019–2023

	Culture positive	Culture negative
PCR positive	40.5% (n = 53)	3.0% (n = 4)
PCR negative	14.5% (n = 19)	42.0% (n = 55)

Among ten endogenous endophthalmitis cases, *C. albicans* was cultured twice, while one *S. aureus* and one *S. agalactiae* strain were identified.

With regard to the antimicrobial susceptibility patterns, multidrug resistance was rare. Indeed, only a single methicillin-resistant *S. aureus* (MRSA) strain was observed. All Gram-positive bacteria remained susceptible to vancomycin.

Overall, bacterial culture positivity was more common in post-injection (60%) and post-cataract (55%) endophthalmitis than in endogenous cases (20%; *p* = 0.06, Fisher’s exact test).

The time to microbiological processing (*i.e*., precise time between sampling and initiation of laboratory processing) was available in 44 cases and was on average 9.5 ± 9.6 h (range 0.3–46.4). No correlation was found between time-to-microbiological-processing and positive culture (*p* = 0.6, r = 0.09) or PCR result (*p* = 0.9, r = –0.02, Spearman).

## Discussion

This study provides an epidemiological assessment of the pathogen spectrum giving rise to endogenous and exogenous postprocedural endophthalmitis in patients presenting to tertiary eye care centers in Saarland, southwest Germany. Differences between diagnostic tools are addressed.

Infectious endophthalmitis mostly occurs exogenously. Eye surgery is reported to account for the largest proportion of postprocedural exogenous endophthalmitis cases [4–7]. However, in this study, endophthalmitis after intravitreal injection was considerably more frequent than after cataract surgery. In half of the herein reported vitreous aspirate samples, bacteria were grown in culture, which is in line with recent literature [8]. Specifically, *S. epidermidis*, which belongs to the physiological flora of the human skin and ocular surface [9], is described as the most common species to cause endophthalmitis [10, 11]. Loukovaara *et al.* reported *S. epidermidis* as the causative agent in four out of 17 cases of endophthalmitis after vitreoretinal surgery [8]. Blom *et al.* observed positive endophthalmitis cultures in 76% of postcataract and postinjection eye samples, in which *S. epidermidis* was the most frequently detected pathogen [5]. Other bacteria such as *S. aureus* and *E. faecalis* occurred also frequently, which is in agreement with previous reports [12]. Indeed, *E. faecalis* represented 20.8% of culture-identified microorganisms in eight South Korean departments from 2004 to 2010. *S. aureus* is also regularly reported in endophthalmitis samples [12–15]. The considerable proportion of post-injection cases due to staphylococci in this cohort likely reflects the increasing volume of intravitreal therapies in routine retinal care. *S. epidermidis* commonly originates from the patient's periocular skin and conjunctival flora, and thus, it is important to minimize bacterial contamination. Preventing post-injection endophthalmitis relies primarily on strict antisepsis and standardized aseptic techniques. Current guidance emphasizes ocular surface antisepsis as the most consistently supported preventive measure, including adequate contact time and application to the conjunctival sac prior to injection. In addition, droplet contamination precautions (e.g., through staff masking and avoidance of talking) can further reduce the infection risk. Antibiotic prophylaxis before intravitreal injections was not shown to be beneficial, and is thus not routinely recommended [16].

A combination of intravitreally applied vancomycin and ceftazidime is the most frequently employed antibiotic treatment to treat infectious endophthalmitis in Germany [11]. All Gram-positive bacterial pathogens detected in our cohort remained susceptible to vancomycin, whereas the absence of Gram-negative pathogens in most samples might question the routine use of ceftazidime in all patients. Moreover, ceftazidime is not the first treatment of choice to treat *H. influenzae*, which was the main Gram-negative pathogen in our cohort. Grandi *et al.* provide a detailed description pertaining to the evolution of antibiotic resistance between 1988 and 2017 [12]. Of note, the risk of specific, albeit rarely occurring adverse events due to antibiotics must also be considered in treatment decisions, such as hemorrhagic occlusive retinal vasculitis, a delayed hypersensitivity reaction to vancomycin [17].

*Candida* spp. is known as a major cause of endogenous endophthalmitis, as it is frequently a complication of previous systemic candidemia [6]. Importantly, blood cultures should always be drawn if endogenous endophthalmitis is suspected. However, routine fundoscopy screening of candidemia patients for ocular candidiasis remains controversial, as the therapeutic impact of detecting chorioretinal signs is uncertain [18, 19]. In our cohort, fungal causes of endogenous endophthalmitis were present, but accounted for a minority of cases.

Microscopy after Gram staining and bacterial culture represent the current standard reference method for pathogen identification in endophthalmitis [20, 21]. However, there is a considerable proportion of endophthalmitis samples that remain culture-negative. This may be explained by prior antimicrobial therapy before referral, low microbial load, limited sample volume, or the presence of fastidious and biofilm-associated organisms not readily detected by routine culture. In addition, a minority of cases may have represented severe sterile postoperative inflammation mimicking infection. These findings highlight the diagnostic limitations of culture alone and support the use of complementary molecular techniques such as PCR and sequencing, which might translate into improved outcomes. In our study, the most sensitive approach was a combination of microscopy, culture, and PCR, as each single technique would have missed some infections. In oligobacterial infections with low bacterial loads, culture is presumably more sensitive than PCR, which may also be due to the very small sample volume used for PCR. Lastly, the time taken to process the vitreous aspirates in the microbiological laboratory does not appear to influence the results, although our conclusion is limited by the lack of precise timeline data in 65.1% of cases. Of note, some bacteria detected in this cohort might also reflect contamination rather than true infection, in particular those that might occur as physiological oropharyngeal or cutaneous colonization, e.g. *N. subflava, K. rhizophila* and different *Streptococcus* spp. This is underscored by the observation that such bacteria were frequently present in samples with more than one bacterium being concurrently detected—an uncommon finding in endophthalmitis, which is typically caused by a single pathogen.

The main limitation of this study is its retrospective nature. A larger sample size might have helped to detect differences in the pathogen spectrum between different patient groups, e.g., those with endophthalmitis after intravitreal injection as compared to specific types of surgery. Owing to a different microbiological work-up, anterior chamber aspirates were also not considered for this analysis, which might have impacted on the pathogen distribution. Also, the included postprocedural endophthalmitis patients were initially operated in several different eye centers, and the majority of patients were only admitted to the two study hospitals once they developed clinical signs of endophthalmitis. Thus, prophylactic and other procedures as well as previous antibiotic treatment strategies may have varied across these centers. For the same reason, we could not consistently assess the time interval between the ophthalmological procedure and the subsequent onset of endophthalmitis, which might have helped to differentiate early-onset inflammatory or toxic reactions from ‘true’ endophthalmitis cases. This could have partially helped explaining the ‘false-negative’ microbiological results. Hence, there is a need for prospective studies that equally assess clinical ophthalmological aspects, microbiological characteristics and infectious disease conditions. Such studies could also delineate whether e.g., the low amount of Gram-negative pathogens observed in our study can be explained by setting-specific idiosyncrasy or represents a lasting epidemiological shift in Western Europe.

We conclude that *S. epidermidis* continues to be the most common pathogen in endophthalmitis patients. Microbiological culture had the highest detection rate, followed by PCR and Gram stain. Combined approaches that include culture and molecular testing yield significantly improved detection results compared to any single diagnostic technique, which is thus highly recommended. Gram-negative pathogens were uncommon in this cohort, which may inform empiric treatment considerations in similar Western European postprocedural settings. However, therapeutic decisions should continue to account for individual clinical context and local epidemiology. Further prospective multicenter studies are warranted to validate whether the low proportion of Gram-negative pathogens observed here reflects a broader epidemiological trend. Close collaboration between ophthalmology and clinical microbiology/infectious diseases is warranted to further improve diagnostics and to tailor antimicrobial treatment procedures.

## Data Availability

Data insight can be obtained from the corresponding author upon reasonable request.
